# A comprehensive combined dataset on *Hibiscus and Tea plant leaf disease* images for classifications

**DOI:** 10.1016/j.dib.2025.112357

**Published:** 2025-12-11

**Authors:** Md Masum Billah, Saifuddin Sagor, Mohammad Shorif Uddin

**Affiliations:** a Computer Science & Engineering, Daffodil International University, Dhaka, Bangladesh; b Computer Science & Engineering, Jahangirnagar University, Savar, Dhaka, Bangladesh

**Keywords:** Artificial intelligence, Image processing, Computer vision, Data science, Machine learning, Hibiscus plant leaves, Tea plant leaves

## Abstract

In this study, we present a combined image dataset created from two distinct plant species: Hibiscus and Tea leaf. The dataset consists of high-resolution images of leaves from both species, captured using a SONY α7 II DSLR camera and a OnePlus 7T lubricant Tea Leaf dataset includes images categorized into five disease classes: Algal Leaf Spot, Brown Blight, Grey Blight, Red Leaf Spot, and Healthy, while the Hibiscus Leaf dataset includes images labeled across eight conditions, including citrus spot, fungal infection, mild edge damage, and healthy foliage. To ensure balanced representation and address class imbalances, extensive data augmentation techniques—such as flipping, rotation, zooming, shifting, noise addition, and brightness adjustment—were applied, resulting in a total of 1,413 combined original images and 13,000 augmented images. The ConvNextTiny deep learning model was fine-tuned on this combined dataset to classify the various leaf conditions, achieving an overall accuracy of 96%. This demonstrates the model's robust performance and high discriminatory power across the diverse set of leaf diseases and conditions. This experiment highlights the utility of combining multiple plant species into a single dataset and utilizing a lightweight yet effective model like ConvNextTiny for plant disease classification. The resulting dataset, along with the model and training scripts, is publicly available to facilitate further research in plant pathology, computer vision, and smart farming applications, enabling more accurate and efficient early-stage disease detection for both Hibiscus and Tea plants.

Specifications Table

**Table:** 

Subject	Computer Sciences
Specific subject area	Artificial Intelligence, Image Processing, Computer Vision, Data Science, Machine Learning, Hibiscus Plant Leaves.
Type of data	Image
Data collection	We have created a combined dataset of Tea (Camellia sinensis) and Hibiscus leaves for plant disease classification. The TeaLeafBD dataset includes 248 high-resolution images (560 × 420 pixels) of tea leaves, categorized into five disease-related classes: Algal Leaf Spot, Brown Blight, Grey Blight, Red Leaf Spot, and Healthy. Images were captured using a SONY α7 II DSLR camera in diverse tea-growing regions of Bangladesh, including Sylhet and Srimangal. Data augmentation techniques such as flipping, rotation, and zooming were applied to enhance model generalization. The Hibiscus Leaf dataset consists of 1165 high-resolution images (560 × 420 pixels) categorized into eight classes: citrus spot, early mild spotting, fungal infection, mild edge damage, senescence, slight disease symptoms, leaf wrinkling, and healthy foliage. Augmentation techniques were similarly applied to both datasets, resulting in a total of 13,000 images. The data collection process was carried out from April 2024 to October 4, 2025, under real-world conditions that included challenges such as inclement weather (rain, overcast skies) and environmental factors like dust and debris. Despite these challenges, all images were carefully reviewed and curated to maintain high quality and consistency. The combined dataset, collected with ethical approval from Daffodil International University, provides a diverse and reliable set of images for developing robust and accurate plant disease classification models.
Data source location	Town/City/Region: Daffodil Smart City Country: Bangladesh Town/City/Region: Sylhet Country: Bangladesh
Data accessibility	Repository name: Mendeley Data Data identification number: 10.17632/5bzy89brkv.4 Direct URL to data: https://data.mendeley.com/datasets/5bzy89brkv/4 GitHub Repository: https://github.com/billahmasumcu/Data_Augmentation_Hibiscus_Tea-Leaf-Disease.git
Related research article	https://ieeexplore.ieee.org/abstract/document/11034939

## 1 Value of the Data

• The combined Hibiscus and Tea leaf datasets offer a high-quality, diverse set of images that enable the development of machine learning models for early disease detection in plants. These models assist farmers and researchers in identifying plant health issues efficiently, facilitating timely interventions, and reducing crop damage. The dataset’s ability to identify a wide range of symptoms ensures more comprehensive disease management strategies.
• By automating disease detection, the datasets support the development of advanced agricultural technologies. Automated systems trained on this data reduce the reliance on manual labor for monitoring plant health, making disease detection faster and more accurate. This results in healthier crops, fewer missed outbreaks, and reduced labor costs, contributing to more sustainable agricultural practices.
• The datasets feature a diverse range of leaf conditions—healthy and diseased—ensuring that models trained on them can detect multiple plant health issues across different environmental conditions. This variety is crucial for creating models that perform well in the real world, where plant conditions can vary significantly across regions.
• Early detection of plant diseases through models trained on these datasets allows farmers to implement targeted treatments, reducing the need for excessive pesticide use. This approach promotes environmentally friendly farming practices by minimizing the ecological impact of pesticide overuse, aligning with sustainable agriculture goals.
• Both datasets are publicly available, encouraging worldwide collaboration among researchers, developers, and farmers. By promoting reproducible research, the datasets support innovation in plant health monitoring, machine learning, and AI applications across various domains, including agriculture, plant pathology, and digital forensics.
• In addition to agricultural applications, the high-resolution images in these datasets hold potential for use in non-agricultural fields such as digital forensics, anomaly detection, and image quality enhancement research. The detailed visual features of the datasets can support advancements in AI and computer vision in diverse sectors beyond plant health monitoring.
• This dataset uniquely provides multiple plant leaf disease (Hibiscus + Tea) images from real-field conditions in Bangladesh, covering diverse disease classes and addressing the lack of diversification of real field plant disease data.

## 2 Background

Hibiscus plants, like many crops, are vulnerable to various diseases that significantly affect both their yield and leaf quality [[13], [14], [15]]. Early detection is crucial to prevent crop loss, yet traditional methods relying on expert visual inspections are slow, error-prone, and impractical for large plantations. To address this, we compiled a specialized collection of Hibiscus and Tea on images into a single dataset, which includes 1165 original high-resolution Hibiscus images and 248 original high-resolution Tea leaf images. These images are categorized into disease classes, such as Healthy, Citrus Spot, Early Mild Spotting, Fungal Infection, Mild Edge Damage, Senescent, Slightly Diseased, and Wrinkled Leaf for Hibiscus, and Algal Leaf Spot, Brown Blight, Grey Blight, Red Leaf Spot, and Healthy for Tea. Advanced data augmentation techniques were applied to the combined dataset, resulting in a total of 13,000 augmented images, which enhanced the model's ability to generalize across different plant health conditions. The ConvNextTiny model was tested on this combined dataset, achieving a classification accuracy of 96 %. This dataset provides a valuable resource for developing automated plant health monitoring systems using deep learning, offering essential benchmarks for researchers and developers in plant pathology, precision agriculture, and AI-driven crop monitoring [[9], [10], [11]] (Table 1).

**Table 1:** Comparison with existing datasets.

Ref.	Name of Data	Size of dataset	Source of dataset
[1]	Roselle Morphological Characterization Dataset	4 varieties × 12 plants × 4 replications = 192 samples (approx.)	National University of Asunción, Faculty of Agricultural Sciences & Research Center (Paraguay)
[2]	Tea Leaf Disease Classification Dataset	6 categories of tea leaf diseases (Algal Spot, Brown Blight, Gray Blight, Healthy, Helopeltis, Red Spot)	Collected from tea plantations (region not specified)
[3]	Tropical Flower Dataset: Seven Species from Bangladesh	4319 images	Captured with Redmi Note 11 smartphone in Dhaka Division, Bangladesh
[4]	BDMediLeaves	2029 original images + 38,606 augmented images	Collected in natural lighting from various regions in Dhaka, Bangladesh
[5]	Radish Leaf Disease Dataset	2801 images	Collected from vegetable fields in Bangladesh
[6]	Chamomile & Hibiscus-Based Antidiabetic Drink Study Dataset	6 experimental groups (G0–G5), sample size not specified	In vivo study with herbal extract-based peach drinks (lab-prepared)
[7]	Tea Leaf Dataset (Sylhet)	3330 images of tea leaves categorized into four classes: Red Rust, Brown Blight, Grey Blight, and Healthy Leaves.	Sylhet tea plantations, Bangladesh
[8]	Tea Leaf Disease Dataset (Bangladesh)	4500 high-resolution images of tea leaves, categorized into five classes: Algal Leaf Spot, Brown Blight, Grey Blight, Healthy Leaf, and Red Leaf Spot.	Tea plantations across Bangladesh

## 3 Data Description

**Fig. 1:**
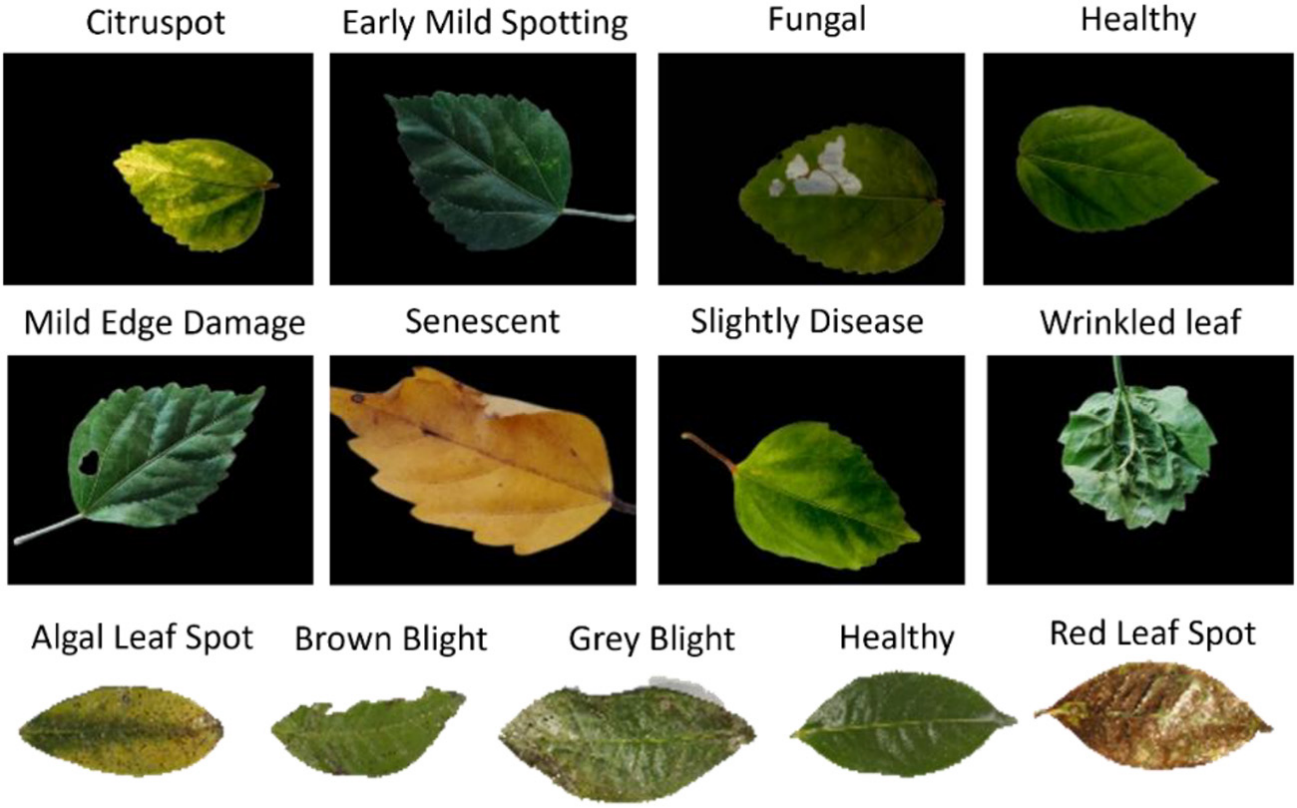
Number of sample images in each class.

The combined Hibiscus and Tea Leaf Diseases Classification Dataset consist of 1165 original Hibiscus leaf images and 248 original Tea leaf images, collected from diverse regions across Bangladesh. These images are categorized into multiple disease-related classes, with the Hibiscus dataset covering categories like healthy, citrus spot, early mild spotting, fungal infection, mild edge damage, senescent, slightly diseased, and wrinkled, and the Tea leaf dataset including classes such as Algal Leaf Spot, Brown Blight, Grey Blight, Red Leaf Spot, and Healthy. Data augmentation techniques were applied to address class imbalances and enhance the model’s generalization capabilities, resulting in a total of 13,000 augmented images. The images were captured using a OnePlus 7T smartphone and a SONY α7 II DSLR camera, with variability in framing, zoom levels, and lighting conditions to reflect real-world All images were acknowledged by plant pathology experts, and the dataset’s practical value for farmers is highlighted through potential mobile-based disease detection applications. The class-wise distribution of images is depicted in Fig. 1, whereas Table 2 summarizes the disease categories for both Hibiscus and Tea leaf datasets.

**Table 2:** Overview of disease by Hibiscus and Tea leaf class.

Class	Description	Visualization
Citruspot	The Citruspot leaf is broadly ovate in shape, with a rounded base and a slightly pointed apex. The margin is mildly undulated, and the surface is smooth and slightly glossy. It displays a clear pinnate venation pattern. The color is generally healthy green, though slight pigmentation variation is present, which may indicate early-stage Citruspot infection	
Early Mild Spotting	Cordate leaf with a rounded base and slightly notched apex. Margin is irregularly serrated; surface smooth and matte. Clear pinnate venation with prominent midrib. Chlorotic patches and darker green zones indicate early mild spotting symptoms. Petiole is visible; leaf structure remains intact.	
Fungal	The leaf in the image is infected by a fungus, showing yellowing patches, dead spots, and uneven coloring mostly near its veins and central rib. While the edges and shiny surface look normal, the damage isn’t even, suggesting the fungus is starting to take hold and affecting how well the leaf can carry out photosynthesis and stay healthy.	
Healthy	The leaf in the image looks healthy, with a nice, even green color and smooth edges. You can clearly see the veins, and there are no signs of yellowing, spots, or damage from disease. Overall, the leaf appears strong and in great condition.	
Mild Edge Damage	The leaf has a little bit of damage around the edges, with some small scrapes and slight color changes near the tips. The veins look fine, and the rest of the leaf seems healthy without any serious spots or yellowing. It looks like the leaf is just beginning to feel some mild stress, maybe from a bit of rough handling or a small infection.	
Senescent	A senescent leaf exhibiting chlorophyll degradation, resulting in a yellow pigmentation due to the dominance of carotenoids. The lamina shows uniform discoloration with minor necrotic speckling, indicating late-stage foliar aging. Marginal desiccation is visible, accompanied by a slight loss of turgor pressure	
Slightly Diseased	A leaf presenting early-stage pathogenic stress, with localized discoloration and subtle chlorotic patches. The venation remains structurally intact, although minor irregularities suggest incipient biotic or abiotic interference. Surface texture appears slightly uneven, indicating potential stomatal dysfunction or fungal infiltration.	
Wrinkled leaf	Leaf samples displaying pronounced laminar deformation characterized by surface wrinkling and abnormal venation patterns. The morphological irregularities suggest compromised cell turgor and uneven mesophyll development. Such wrinkling is typically associated with environmental stress, nutrient deficiency, or viral phytopathology.	
Algal Leaf Spot	The image depicts a tea leaf infected with Algal Leaf Spot, characterized by reddish-brown circular lesions caused by the parasitic alga Cephaleuros virescens. These lesions often result in chlorosis, reducing the leaf's photosynthetic capacity. The affected leaf surface appears slightly glossy with localized discoloration. Such visual markers are critical for enabling early disease detection in automated plant health monitoring systems.	
Brown Blight	The tea leaf exhibits symptoms of Brown Blight, marked by dark brown, irregular lesions primarily along the leaf margins. The disease is caused by the fungal pathogen Colletotrichum camelliae, which induces tissue necrosis and leaf curling. These distinct morphological features are critical for early-stage recognition in automated plant disease detection systems.	
Grey Blight	The tea leaf displays symptoms of Grey Blight, characterized by greyish-brown necrotic lesions concentrated near the leaf tips and edges. The disease is caused by the fungal pathogen Pestalotiopsis theae, which leads to progressive tissue damage and leaf curling. These visual indicators are critical for automated disease detection systems in tea cultivation environments.	
Healthy	The image depicts a healthy tea leaf, exhibiting deep green coloration, a smooth surface, and no visible signs of disease, pest damage, or discoloration. The leaf is structurally intact and represents the ideal reference state of tea foliage, serving as the ground truth class in disease classification tasks.	
Red Leaf Spot	The image shows a tea leaf affected by Red Leaf Spot, characterized by reddish-brown discoloration across the surface. Irregular lesions and spots are clearly visible, indicating possible fungal or bacterial infection. This type of leaf is commonly used in datasets for detecting and classifying tea plant diseases [12].	

**Fig. 2:**
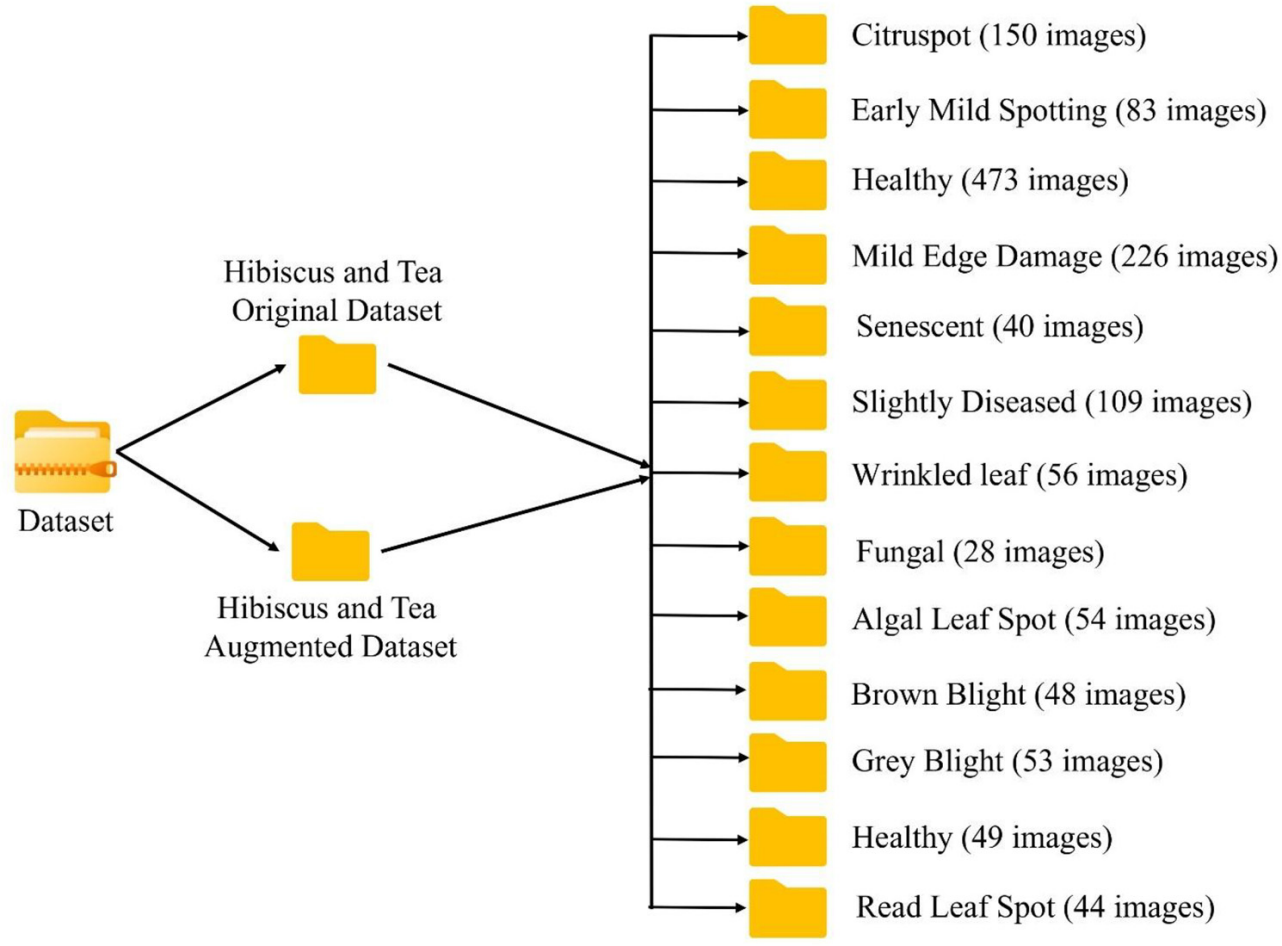
Folder structure of the combined Hibiscus and Tea leaf disease dataset.

Fig. 2 presents the folder structure of the combined Hibiscus and Tea Leaf dataset. The dataset is organized into disease-specific categories, with each folder containing images of leaves classified based on their health condition. The Hibiscus Leaf dataset includes folders for various disease categories such as Citrus Spot (150 images), Early Mild Spotting (83 images), Healthy (473 images), Mild Edge Damage (226 images), Senescent (40 images), Slightly Diseased (109 images), Wrinkled Leaf (56 images), and Fungal (28 images). The Tea Leaf dataset includes folders for Algal Leaf Spot (54 images), Brown Blight (48 images), Grey Blight (53 images), Healthy (49 images), and Red Leaf Spot (44 images). The combined dataset totals a diverse range of images, offering a rich resource for plant disease classification and monitoring.

Table 3 presents the statistics for the Hibiscus and Tea leaf datasets. It outlines the number of original images and the number of augmented images for each disease class. The Hibiscus Leaf Dataset includes a total of 1413 original images across various categories: Citrus Spot (150 images), Early Mild Spotting (83 images), Fungal Infection (28 images), Healthy (473 images), Mild Edge Damage (226 images), Senescent (40 images), Slightly Diseased (109 images), Wrinkled Leaf (56 images), Algal Leaf Spot (54 images), Brown Blight (48 images), Grey Blight (53 images), Healthy (49 images), and Red Leaf Spot (44 images). For each class, data augmentation techniques were applied, resulting in a total of 13,000 augmented images across the dataset, ensuring balanced representation for improved model training and performance.

**Table 3:** Statistics of Hibiscus and Tea leaf datasets.

Class Name	Number of original images (Before Augmentation)	Number of augmented images (After Augmentation)
Citruspot	150	1000
Early Mild Spotting	83	1000
Fungal	28	1000
Healthy	473	1000
Mild Edge Damage	226	1000
Senescent	40	1000
Slightly Diseased	109	1000
Wrinkled	56	1000
Tea Algal Leaf Spot	54	1000
Tea Brown Blight	48	1000
Tea Grey Blight	53	1000
Tea Healthy	49	1000
Tea Red Leaf Spot	44	1000
Total	1413	13,000

## 4 Experimental Design, Materials and Methods

### 4.1 Experimental design

To enable accurate disease classification for both Hibiscus and Tea leaf using deep learning, a well-structured preprocessing pipeline was employed. This pipeline involved data cleaning, augmentation, and dataset partitioning. Initially, redundant or low-quality images were removed to ensure dataset integrity. The dataset was then enhanced through various augmentation techniques, including flipping, rotation, zooming, noise addition, height shifting, and brightness adjustment, introducing geometric, photometric, and positional variability. These transformations increased sample diversity while preserving disease-specific visual patterns, thus improving the model’s robustness to changes in orientation, scale, illumination, and position during inference. Following augmentation, the dataset was partitioned into three subsets: 70 % for training, 15 % for validation, and 15 % for testing, ensuring sufficient diversity for effective learning and unbiased performance evaluation. This pipeline provides a reliable and reproducible foundation for benchmarking deep learning models for plant disease classification tasks and contributes to the development of intelligent, automated plant health monitoring systems.

### 4.2 Materials (Camera specification)

Images in the dataset were captured using a SONY ALPHA 7 II DSLR camera, which features a 24.3-megapixel full-frame (35 mm) CMOS sensor with a sensor size of 1.0 inch. To ensure a diverse range of perspectives and visual characteristics, multiple lenses and shooting modes were utilized. The main wide-angle images were captured using a 28 mm focal length, while portrait, telephoto, and landscape modes used 50 mm, 70 mm, and 35 mm respectively. This variation in focal lengths allowed for capturing Hibiscus leaf images under different spatial scales and framing conditions, enriching the dataset’s variability and enhancing its applicability to robust computer vision tasks. Additionally, images were captured using a OnePlus 7T smartphone, which features a 48 MP primary camera with an f/1.7 aperture and a 26 mm focal length for high-resolution imaging. The OnePlus 7T offers versatility in image capture, providing supplementary perspectives that complement the high-quality DSLR shots, enhancing the dataset’s diversity and its suitability for deep learning applications in plant disease classification.

### 4.3 Methods

**Fig. 3:**
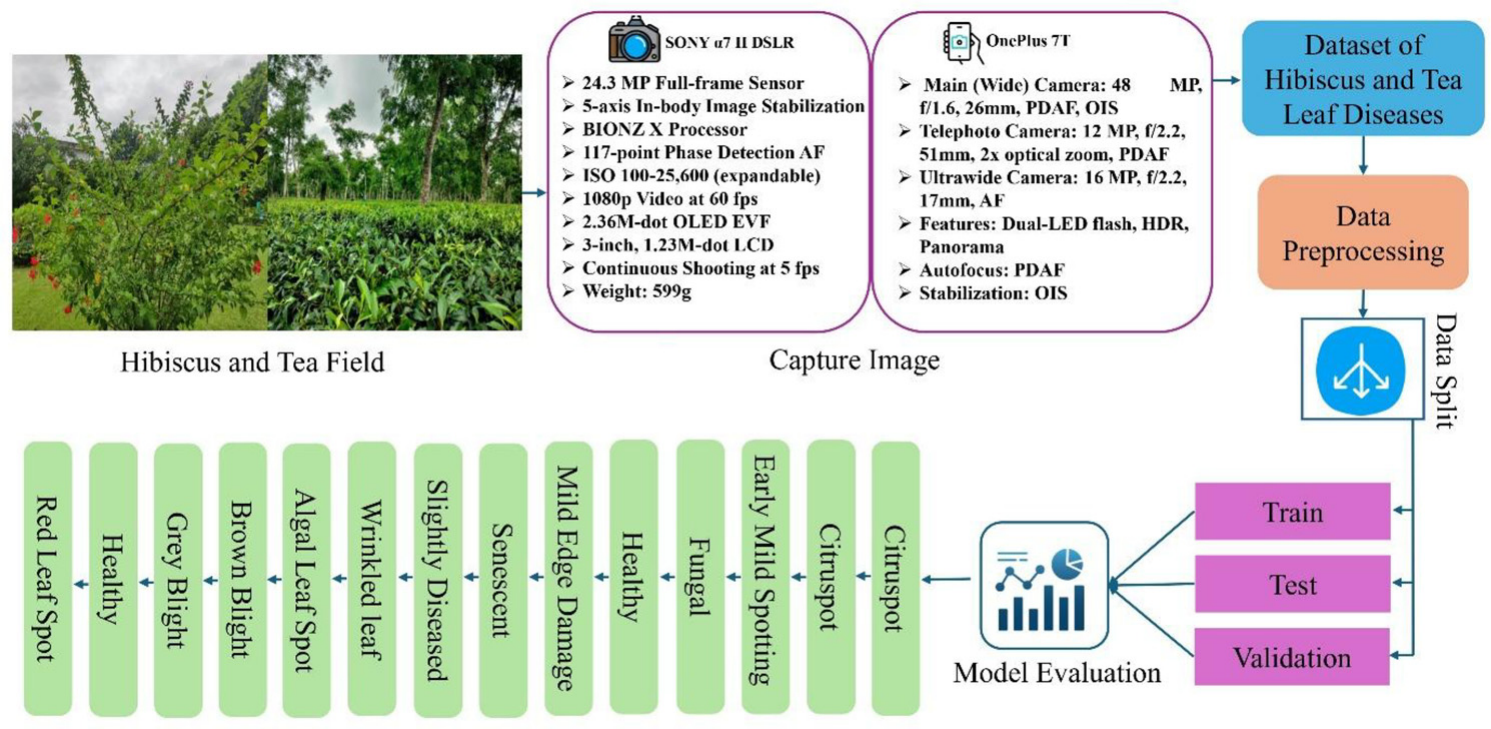
Workflow of the experimental design for the detection and classification of the Hibiscus and Tea leaf disease.

Fig. 3 shows the experimental design for detecting and classifying diseases in Hibiscus and Tea leaf. Images were captured from a Hibiscus and Tea field using two devices: the SONY α7 II DSLR camera, featuring a 24.3 MP full-frame sensor, 5-axis in-body image stabilization, BIONZ X processor, and 117-point phase detection autofocus for high-resolution, stable, and sharp images; and the OnePlus 7T smartphone, equipped with a 48 MP main camera, a 12 MP telephoto lens, optical image stabilization, HDR, and Dual-LED flash for versatile image capture under varying conditions. These images were processed into the Dataset of Tea and Hibiscus Leaf Diseases, followed by data augmentation to enhance diversity. The dataset was then partitioned into Training, Testing, and Validation subsets to evaluate the deep learning model's performance in classifying plant diseases. This workflow supports the development of robust models for automated plant health monitoring systems, ensuring effective detection and classification of leaf diseases in real-world agricultural environments.

**Fig. 4:**
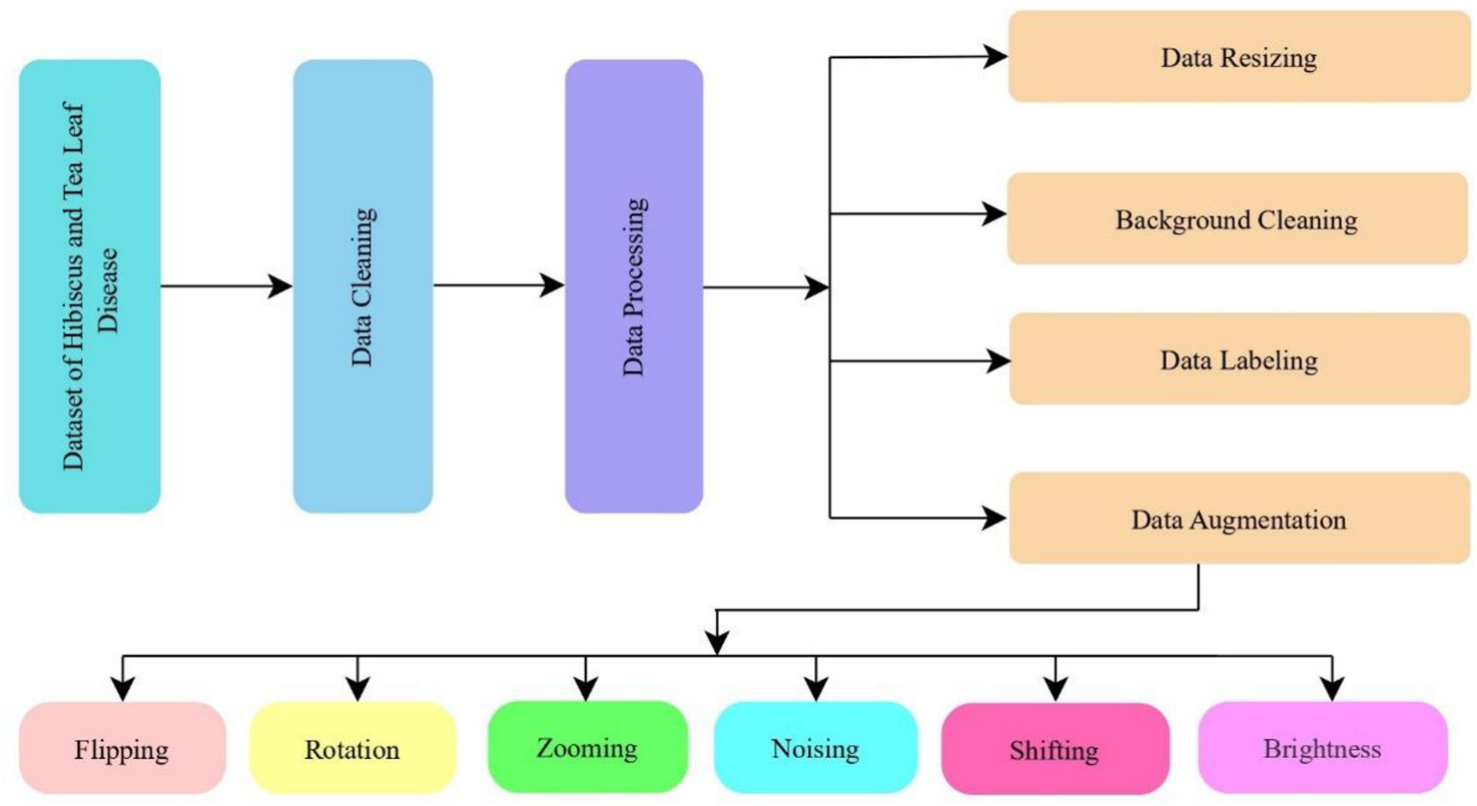
Pre-processing stages of the proposed deep learning model in the detection of Hibiscus and Tea leaf diseases.

Fig. 4 Presents the pre-processing stages used in preparing the Hibiscus and Tea leaf datasets for deep learning-based disease detection. The process begins with the Dataset of Hibiscus and Tea Leaf Disease, followed by Data Cleaning, which ensures the removal of any redundant or low-quality images. Data Processing includes several critical steps such as Data Resizing, Background Cleaning, Data Labeling, and Data Augmentation. The augmentation process involves various techniques, including Flipping, Rotation, Zooming, Noising, Shifting, and Brightness adjustments, which enhance the diversity of the dataset by introducing geometric, photometric, and positional variations. These pre-processing steps ensure the dataset is well-prepared for training, enhancing model generalization and performance in real-world disease classification tasks.

### 4.4 Data augmentation

Data augmentation enhances model optimization and generalizability by generating new data samples from existing ones. This method effectively expands the available training data without requiring additional data collection, which is particularly advantageous when dealing with limited datasets, such as Hibiscus and Tea leaf images. Overall, data augmentation is critical in improving the accuracy and reliability of machine learning models across various domains. In the context of deep learning,

We deploy several augmentation techniques on Hibiscus and Tea leaf disease classification including flipping, rotation (within the range of −30 to 30 degrees), zooming (factors of 0.8, 1.0, and 1.2), shifting, noise addition, and brightness adjustment (with factors of 0.7 and 1.3). These techniques introduce variations into the dataset, allowing the model to better generalize to unseen samples. As a result, the model becomes more robust in classifying Hibiscus and Tea leaf diseases under varying conditions, such as different leaf orientations, lighting conditions, and camera angles.

**Fig. 5:**
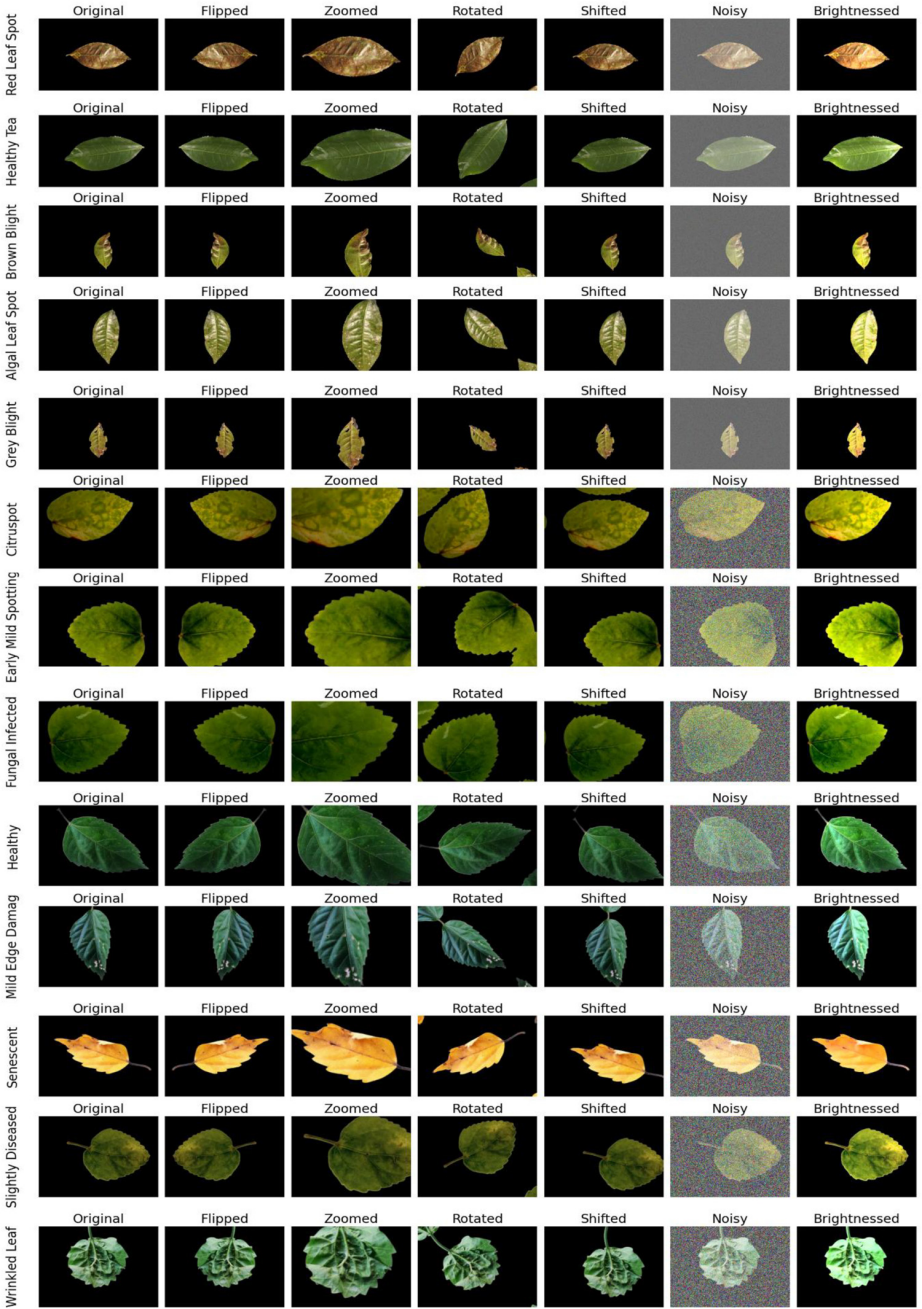
Augmented photos of Hibiscus leaf disease dataset.

Fig. 5 illustrates various data augmentation techniques applied to the Hibiscus and Tea leaf disease dataset. Each row represents a different disease category, including Algal Leaf Spot, Brown Blight, Grey Blight, Healthy Tea, Red Leaf Spot, Citrus Spot, Early Mild Spotting, Fungal Infected, Healthy Hibiscus, Mild Edge Damage, Senescent, Slightly Diseased, and Wrinkled Leaf. For each disease class, the first column shows the original image, followed by images that have undergone different augmentation techniques: Flipping, Zooming, Rotation, Shifting, Noise Addition, and Brightness Adjustment. These transformations introduce geometric, photometric, and positional variations into the dataset, enhancing the model's ability to generalize across diverse real-world conditions and improving its robustness in classifying leaf diseases under different orientations, lighting conditions, and perspectives.

## 5 Results

This section clearly summarizes all model evaluation results, including the main performance metrics, training and validation curves, the confusion matrix, and the classification report.

Table 4 summarizes the performance of the ConvNextTiny model for Hibiscus and Tea leaf disease classification using several classification metrics. The model's performance is evaluated using Precision, Recall, F1-score, Support, and Accuracy for each disease class. The table shows strong performance across all categories, with Fungal (0.99), Healthy Hibiscus (0.95), and Mild Edge Damage (0.99) achieving high precision, recall, and F1-scores. The Senescent class achieved a perfect score (1.00) in all metrics, indicating a flawless classification for this category. The overall Accuracy of the model across all classes is reported as 96 %, showcasing its robust ability to classify both Hibiscus and Tea leaf diseases ofConvNextTiny model in handling a variety of plant disease classification tasks.

**Table 4:** Performance of ConvNextTiny based on classification metrics and accuracy.

Model	Class Name	Precision	Recall	F1-score	Support	Accuracy
ConvNextTiny with data augmentation	Hibiscus Citruspot	0.98	0.93	0.96	150	96 %
	Hibiscus Early Mild Spotting	0.89	0.92	0.90	150	
	Hibiscus Fungal	0.97	1.00	0.99	150	
	Hibiscus Healthy	0.97	0.90	0.93	150	
	Hibiscus Mild Edge Damage	0.99	0.97	0.98	150	
	Hibiscus Senescent	1.00	1.00	1.00	150	
	Hibiscus Slightly Diseased	0.88	0.95	0.92	150	
	Hibiscus Wrinkled leaf	1.00	1.00	1.00	150	
	Tea Algal Leaf Spot	0.93	0.95	0.94	150	
	Tea Brown Blight	0.98	0.97	0.97	150	
	Tea Grey Blight	0.97	0.93	0.95	150	
	Tea Healthy Tea	0.95	0.99	0.97	150	
	Tea Red Leaf Spot	0.99	0.97	0.98	150	

**Fig. 6:**
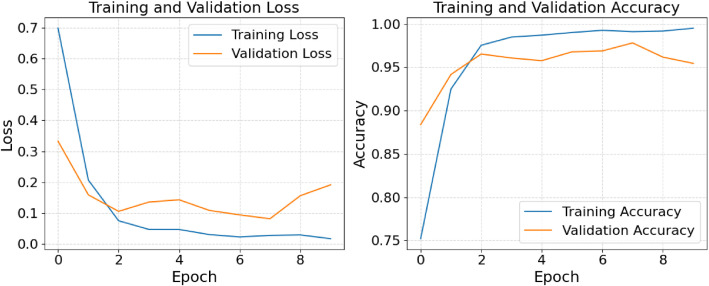
Training and validation accuracy and loss curves for ConvNextTiny with data augmentation.

Fig. 6 shows the training and validation accuracy and loss curves for the ConvNextTiny model applied to the Hibiscus and Tea leaf disease classification task, after data augmentation. On the left, the Training and Validation Loss curve demonstrates a rapid decrease in loss during the early epochs, with the training loss decreasing sharply and stabilizing at a lower value, while the validation loss also decreases but with a slight gap between the training and validation curves, indicating minor overfitting. On the right, the Training and Validation Accuracy curve shows a sharp rise in training accuracy, quickly reaching high performance, while the validation accuracy gradually increases and stabilizes close to 96 %. This pattern indicates that the model is successfully learning and generalizing well to new data, achieving robust performance across both training and validation datasets.

**Fig. 7:**
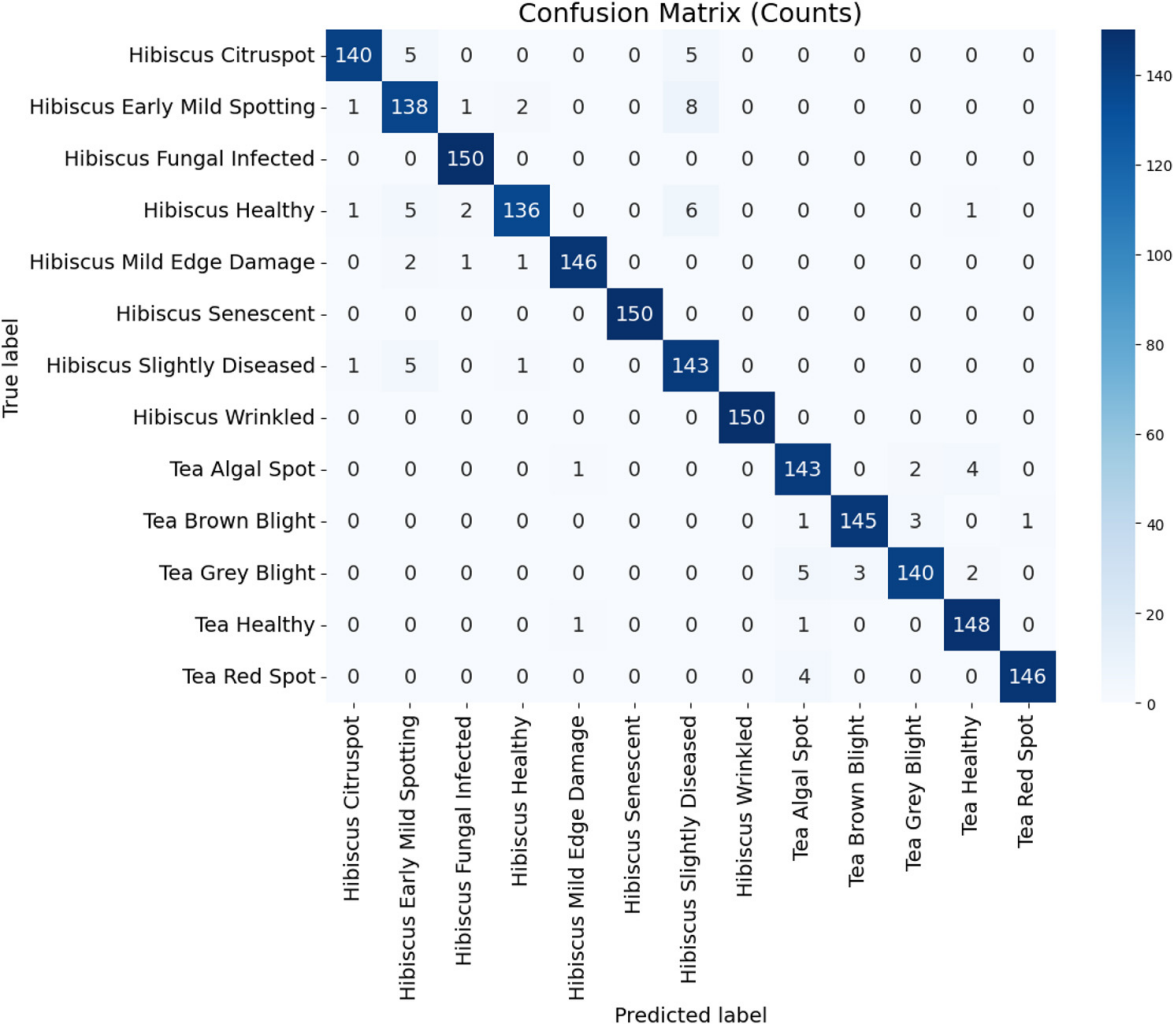
Confusion matrix of ConvNextTiny with validation of training data.

The confusion matrix shows in Fig. 7 that explicitly represents the strong classification performance across all 13 classes with most predictions are correctly aligned along the diagonal for both Hibiscus (8 classes) and Tea leaves (5 classes). While a few Hibiscus classes are minor confusion with visually similar categories, the Tea leaf classes are classified with consistently high accuracy, demonstrating robust overall model performance.

## Limitations

While our work covers a wide range of topics, it isn't without its limitations. Although our dataset is sizable, it doesn't yet capture every possible variation in leaf appearance. Differences in lighting, background scenery, and even leaf orientations could be broader. These gaps may impact the models' performance when applied to real-world conditions. On the bright side, we have no conflicts of interest to disclose; every insight and suggestion presented here originates directly from our experiments and our goal of advancing hibiscus leaf disease detection.

## Ethics Statement

We confirm that the authors have read and followed the ethical requirements for publication in Data in Brief. The dataset was collected in collaboration Daffodil International University and Jahangirnagar University, Bangladesh.

## CRediT Author Statement

**Md Masum Billah:** Conceptualization, Data Curation, Methodology, Writing; **Saifuddin Sagor:** Data Curation, Methodology; **Mohammad Shorif Uddin:** Supervision, Writing – Review & Editing.

## Data Availability

Mendeley DataA Real-World Hibiscus and Tea Leaf Image Dataset for Classification (Original data).
